# Spatial Variability of Metals in Surface Water and Sediment in the Langat River and Geochemical Factors That Influence Their Water-Sediment Interactions

**DOI:** 10.1100/2012/652150

**Published:** 2012-08-01

**Authors:** Wan Ying Lim, Ahmad Zaharin Aris, Mohamad Pauzi Zakaria

**Affiliations:** Centre of Excellence for Environmental Forensics, Faculty of Environmental Studies, Universiti Putra Malaysia, 43400 UPM Serdang, Selangor, Malaysia

## Abstract

This paper determines the controlling factors that influence the metals' behavior water-sediment interaction facies and distribution of elemental content (^75^As, ^111^Cd, ^59^Co, ^52^Cr, ^60^Ni, and ^208^Pb) in water and sediment samples in order to assess the metal pollution status in the Langat River. A total of 90 water and sediment samples were collected simultaneously in triplicate at 30 sampling stations. Selected metals were analyzed using ICP-MS, and the metals' concentration varied among stations. Metal concentrations of water ranged between 0.08–24.71 **μ**g/L for As, <0.01–0.53 **μ**g/L for Cd, 0.06–6.22 **μ**g/L for Co, 0.32–4.67 **μ**g/L for Cr, 0.80–24.72 **μ**g/L for Ni, and <0.005–6.99 **μ**g/L for Pb. Meanwhile, for sediment, it ranged between 4.47–30.04 mg/kg for As, 0.02–0.18 mg/kg for Cd, 0.87–4.66 mg/kg for Co, 4.31–29.04 mg/kg for Cr, 2.33–8.25 mg/kg for Ni and 5.57–55.71 mg/kg for Pb. The average concentration of studied metals in the water was lower than the Malaysian National Standard for Drinking Water Quality proposed by the Ministry of Health. The average concentration for As in sediment was exceeding ISQG standards as proposed by the Canadian Sediment Quality Guidelines. Statistical analyses revealed that certain metals (As, Co, Ni, and Pb) were generally influenced by pH and conductivity. These results are important when making crucial decisions in determining potential hazardous levels of these metals toward humans.

## 1. Introduction

Heavy metal pollution has become a major concern, especially in industrialized countries due to their toxicity, persistence, and bioaccumulative nature. Heavy metals are not only regarded as natural trace components of the aquatic environment but are also commonly known as environmental pollutants particularly receiving input from anthropogenic activities. Their occurrences not only originated from natural factors such as the weathering of soils and rocks but also from anthropogenic input such as industrial waste, agricultural and mining activities [1–3]. Metals undergo numerous changes in their speciation due to dissolution, precipitation, sorption, and complexation phenomena when discharged into a water body [4–6]. When metals enter the environment, they will distribute between the aqueous phase and the suspended sediments during their transport [7]. Metals tend to be assimilated in sediment with organic matter, Fe/Mn oxides, sulphide, and clay thus forming several reactive components, which are harmful to the environment [5, 8]. Hence, sediment is always regarded as the potential reservoir for metals and plays an important role in adsorption of dissolved heavy metals [5, 8]. Under different physical and chemical conditions, metals in sediment may leach out into the water column as free ions. In turn, contaminated sediments also act as sources of heavy metals when released into the river water. Metals' concentration in river water can be regarded as a good indicator of the river contamination. Metal ions can be either an essential nutrient or toxic to living organisms [4]. When the metals' concentration exceeds standard permissible limits, it would have toxic effects on living organisms and cause negative impact on lower life forms. For example, transition metals such as copper (Cu), zinc (Zn), cobalt (Co), manganese (Mn), nickel (Ni), and iron (Fe) are essential nutrients; however, they may be toxic if in high concentration. Lead (Pb), cadmium (Cd), mercury (Hg), chromium (Cr), and arsenic (As) are toxic to living organisms even at low concentrations [9]. Some of the metals such as Pb, Cd, Hg, and As are well known as global contaminants and are listed as the most hazardous inorganic contaminants on the EPA Hazardous Substance Priority List which have detrimental effect on the health of people and ecology [10]. They are freely dissolved in water, extremely toxic, and have the significant impact on environmental and public health concern [11, 12]. When these metals enter the food chain through phytoplankton and are biomagnified in aquatic organisms, they would pose a potential risk to human health [9, 13].

Numerous studies were conducted to assess and establish the extent of metal contamination in the rivers. Most riverine studies dealing with metals associated with water, sediment, or biota are concerned with total metal concentration. Elevated total dissolved concentrations of Pb, Cu, Cd, Ni and Zn have been reported in different parts of the world [3, 6, 12, 14, 15]. These metals present in excessive quantities will interfere with many beneficial uses of the river water due to their toxicity. In Malaysia, research projects have been carried out on some of the important rivers, and it has been observed that most of the river's water quality is gradually deteriorating. Examples researches conducted in Malaysia were such as evaluation of the effects of human activities on trace metal's concentration and water quality [16], trace metal's chemistry and effects of monsoon on metal concentration at Besut River [17], application of multivariate statistical techniques to classify the possible source of pollution at selected estuaries [13], and water quality of the Selangor River [18]. Most of these studies were focused on the total metal content in sediments, organisms, plants, and fresh water bodies in order to have a better understanding on the ecotoxicological potential of heavy metals [19–21]. In addition, previous studies indicated that As, Cd, Cu, Cr, Pb, Ni, and Zn were the most common contaminants found in the Straits of Malacca [1, 2, 22, 23]. These metals were mostly a result of industrial wastes and shipping activities along the Straits of Malacca. 

The main objectives of this paper are to determine the spatial variability of elemental content (^75^As, ^111^Cd, ^59^Co, ^52^Cr, ^60^Ni, and ^208^Pb) in river water and sediment and to evaluate the geochemical factors that influence the metals behavior namely sediment-water interaction. The obtained concentrations in water were compared with the Malaysian National Standard for Drinking Water Quality recommended by the Ministry of Health [24]. Meanwhile, metal concentrations in sediment were compared to the Canadian Sediment Quality Guidelines for the Protection of Aquatic Life for freshwater sediment, that is, for Interim Sediment Quality Guideline (ISQG) and Probable Effect Level (PEL) [25] and average shale values [26]. The results obtained could provide benchmark levels, which could be used in the exploration of strategies to protect human health and the ecosystem.

## 2. Materials and Methods

### 2.1. Site Description

The Langat River is essential to the Selangor population and is one of the important freshwater ecosystems in Selangor. Beside providing potable water for drinking purposes, the Langat River also supplies water for manufacturing and agricultural production. Moreover, it provides considerable sources of food and acts as a breeding ground and is a sanctuary for aquatic organisms [27]. The economic value posed by this ecosystem makes its suitable for aquaculture activities, source of food, recreation, nature tourism, and genetic resources. Yet, rapid development also threats the river water quality and bears a direct effect on the ecosystem. From examples, inadequate water management and uncontrolled contaminants discharged from both industries and economic activities had contributed to river pollution [28]. One of the largest steel-making industries in Malaysia was located in proximity to the upstream area and is considered a possible source of metal's pollution. The river has become the main shipping route for these factories to transport their raw material or finished products. Thus, shipping activities were intense within the study area and contributed substantial amount of pollutants into the river.

**Figure 1:**
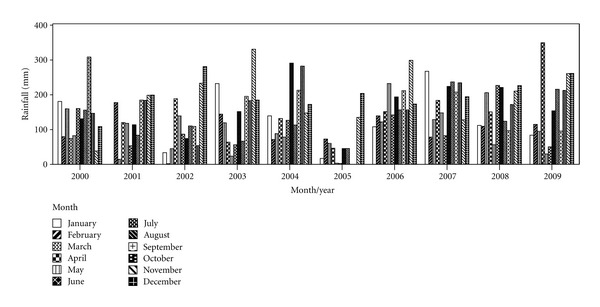
Monthly rainfall data for study area from 2000 to 2009.

**Figure 2:**
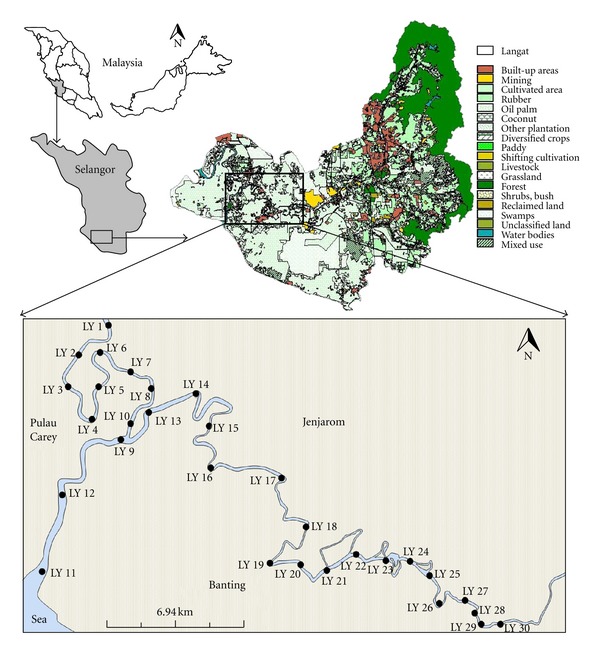
Map of sampling stations in Langat River. Source: JICA and MDGM [29].

The Langat River Basin lies between latitudes 2° 40′ 152′′N to 3° 16′ 15′′N and longitudes 101° 19′ 20′′E to 102° 1′ 10′′E. The basin has a total catchment area of approximately 1815 km^2^ comprising hilly and mountainous terrain and also coastal plain. The main river course is 141 km long and is mostly situated 40 km east of Kuala Lumpur. It is located in the midwestern part of Peninsular Malaysia across two states, Selangor and Negeri Sembilan. The basin consists of two estuaries; one is located at the northeastern side where the river water flows into the Lumut Straits while the other is at the southern side and flows directly into the Straits of Malacca [27]. The study area is located close to the equator and is greatly influenced by two types of monsoon: the northeast monsoon from November to March (rainy season) and the southwest monsoon from April to November (dry season). Langat River receives annual rainfall from 1500 to 2900 mm, which is influenced by the monsoon blows. The basin experiences average temperature of 32°C with relative humidity of 80% annually. The study area experiences high average and uniform annual temperatures, high rainfall, and high humidity. A summary of monthly rainfall from 2000 to 2009, for the study area, is shown (Figure 1). Langat River Basin is underlain by schist, phyllite, and granite rock formation of the Permian Age dominates. In the mountainous area, the bedrock includes Permian igneous rock, pre-Devonian schist, and also phyllite of the Hawthornden Formation [30]. The bedrock near the middle of the basin, buried in the rolling hills, is called the Kenny Hill and Kajang Hill Formation (both metasedimentary deposits) consisting of mostly shale and quartzite. A Quaternary deposit, referred to as alluvium, mainly consists of silt, clay, and sand [31]. There are four types of formation recognized in the Quaternary alluvium, namely, Simpang, Kempadang, Gula, and Beruas Formations, which unconformably overlay on eroded bedrock and grow progressively younger and thicker toward the coastal lowlands [30]. In general, these sediments grade downward from clay to gravel and were deposited in fluvial and shallow marine environments. Geologically, the study area is located in the low flatlands close to the coast, where alluviums are deposited on the bedrocks (Figure 2) [31].

### 2.2. Sampling and Analysis

The sampling was carried out in the raining season (December 2010). A total of 90 water and sediment samples were collected simultaneously at 30 sampling stations along the downstream of Langat River. The exact sampling locations were recorded by the Global Positioning System (GPS) device (Table 1; Figure 2). Triplicate of water and sediment samples were collected and homogenized from each sampling station. The *in-situ* parameters (conductivity and pH) were measured immediately during field work. Water samples were then filtered through 0.45 *μ*m cellulose acetate membrane filter (Whatman Millipore, Clifton, NJ, USA) using a syringe filtration unit. This was done to obtain dissolved metal while avoiding the clogging of spectrometry instrument during analysis. Subsequently, the samples were acidified to pH < 2 with concentrated nitric acid (HNO_3_) in order to prevent precipitation of components such as metal oxides and hydroxides and to retard biological activities [32]. Triplicate of sediment samples were collected by the composite sampling technique using an Ekman grab sampler or plastic scoop. The collected samples were kept in the acid-washed polyethylene bag, immediately brought back to the laboratory, and kept at 4°C prior to analysis [32, 33]. Sediment samples were naturally air-dried in the laboratory until a constant dry weight is obtained. Later, the samples were agitated with a ceramic mortar and pestle and sieved through 2 mm (for physico-chemical parameters) and 63 *μ*m (for heavy metals) mesh screen, respectively. The sieved sediments were stored in the sealed acid-washed polyethylene containers prior to analysis. The conductivity and pH were determined based on electrical measurement using electrodes by using 1 : 2 ratio sediment to double-deionized water [20, 33–35]. For heavy metal's analysis, a fine fraction (<63 *μ*m) sample was chosen as it retained higher metal content compared to other larger sediment sizes and was digested using aqua regia (HCl : HNO_3_ = 1 : 3). In this study, the selected metals (^75^As, ^111^Cd, ^59^Co, ^52^Cr, ^60^Ni, and ^208^Pb) were analyzed using Inductively Coupled Plasma Mass Spectrometry (ICP-MS, ELAN DRC-e, Perkin Elmer). The accuracy of the ICP-MS performance for water analysis was assessed by external standards, which were prepared by diluting the ICP Multi-Element Mixed Standard III (Perkin Elmer) into series of concentrations with the same acid mixture used for sample dissolution. Meanwhile, for sediment analysis, it was assessed by analyzing the standard reference material NIST, SRM 1646a (estuarine sediment). The recovery rate for external standard (water analysis) ranged from 102% to 103% (±5%), while for internal SRM was satisfactory being between 89% and 112%, depending on the element analyzed (Tables 2 and 3). Geochemical and environmental data often show first of all a spatial dependence, and spatially dependent data are not, in general, normally distributed [36]. The dataset should be transformed to render the data normalized and perform common statistical methods water [36]. However, for environmental data, frequently characterized by exceptionally high values that deviate widely from the main body of data, data transformation will not help to approach a normal distribution and can lead to conclusions that are not conservative. Therefore, the statistical methods used for present study are based on assumption that the studied data show a normal distribution [37]. The descriptive statistic was performed to calculate maximum, minimum, mean, standard deviation (SD), and coefficient of variance (CV). ANOVA was applied to test significant differences in heavy metal concentrations at different sampling stations. A post hoc test was performed using the LSD test with the degree of significance at 0.05. Pearson's correlation coefficient (*r*) was performed to see the relationship between physico-chemical parameters and ions in water and sediment. All statistical analyses were performed using PASW Statistics 18 (formerly known as SPSS Statistics 18, or SPSS Base).

## 3. Results and Discussion

### 3.1. Water and Sediment Characteristics

Table 4 shows the descriptive statistic for selected physico-chemical parameters in water and sediment at Langat River. One-way ANOVA showed that the selected physico-chemical parameters varied significantly among the stations (*P* < 0.05). The calculation of the coefficient of variance (CV) for both water and sediment parameters revealed that the data for all parameters varied from the mean which has a CV higher than 90% with the exception of water and sediment pH (Table 3). The variations were more influenced by location, especially in the downstream area. The downstream stations (LY 1–LY 14) were near to estuary and experienced higher level of exposure to seawater (Figure 2). Hence these stations tend to have a higher salinity value and pH value, which is near to neutral or slightly alkaline [33].

### 3.2. Heavy Metals in Water

**Figure 3:**
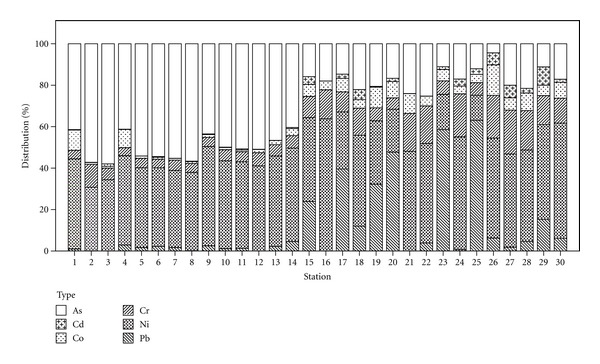
Distribution of metals in water samples from 30 sampling stations at Langat River.

The descriptive statistic for studied metals (As, Cd, Co, Cr, Ni, and Pb) was presented in Table 5. The mean concentrations of the metals were observed in the order of As > Ni > Cr > Pb > Co > Cd (Figure 3). The coefficient of variance (CV) calculated reveals that the metals varied from the mean with the CV value higher than 80%. The high CV implies that measured concentration for all metals varied between stations (*P* < 0.05; ANOVA). By comparing the average concentration of studied metals with the Malaysian National Standard for Drinking Water Quality proposed by the Ministry of Health (MOH) [24], it was found that all metals have an average concentration lower than the standard.

**Figure 4:**
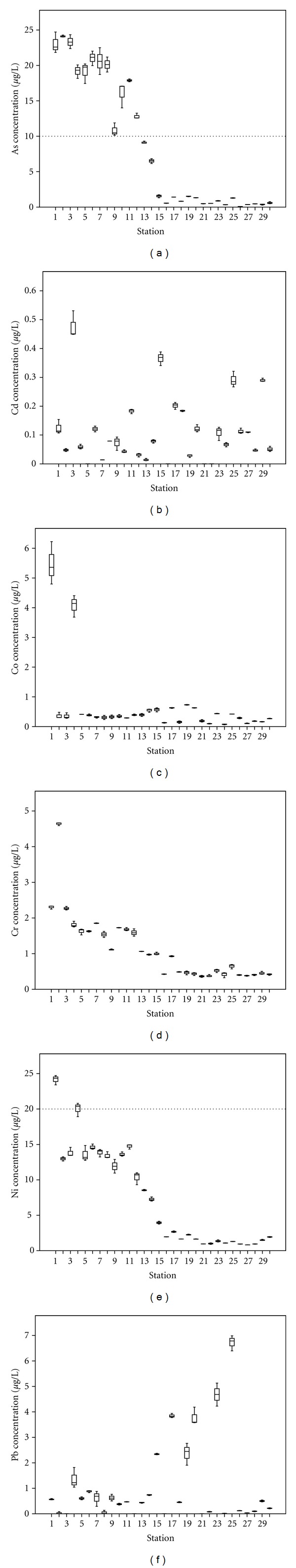
Distribution of each heavy metal concentration (*μ*g/L) along stations in Langat River water samples by comparing with Malaysian National Standard for Drinking Water Quality (MOH).

Overall, the metal's concentrations in water were low, except for As and Pb (*P* < 0.05; ANOVA). The mean concentration of As measured was 8.54 ± 9.15 *μ*g/L. The highest concentration was found in LY 2 (24.71 *μ*g/L) and the lowest in LY 26 (0.08 *μ*g/L). About 40% of the sampling stations were found to exceed the MOH standard (10 *μ*g/L). Elevated content of As from LY 1 to LY 12 may be attributed to the input of arsenical herbicides (monosodium and disodium methylarsonate) and insecticide or tin mining activities within the Langat River Basin. It was also proven by other studies that significant increases of As concentrations in river waters could be considered as a result of pollution from agricultural effluents [22, 23]. The measured mean value for Ni was 7.29 ± 6.75 *μ*g/L, while the highest concentration was found in LY 1 (24.72 *μ*g/L) and the lowest in LY 26 (0.80 *μ*g/L). Only 6% of the sampling stations exceeded the MOH standard (20 *μ*g/L), and the possible sources of Ni may be attributed to inorganic fertilizer, electrolyte nickel plating activities, sewage sludge, and livestock manures [38]. The mean Cr concentration measured was 1.13 ± 0.91 *μ*g/L, the highest concentration was found in LY 2 (4.67 *μ*g/L), and the lowest in LY 21 (0.32 *μ*g/L). There was an increasing trend (*P* < 0.05; ANOVA) of As, Cr, and Ni concentration from upstream (LY 30) to the downstream (LY 1). The metal concentrations increased when moving downward to the Straits of Malacca or Lumut Straits. This is mainly due to the downstream area receiving a significant amount of anthropogenic inputs from the river upstream as it flowed through urban areas, agricultural and industrial area [39, 40]. Meanwhile, river downstream is also intense with human activities especially in manufacture and industrial facilities. The point source input from the industrial zone which was located adjacent to the river increases the frequency of pollution occurrence within this area. Hence, the variation of metals between was more influenced by its location, especially in the downstream region. The similar distribution pattern existed between As, Cr, and Ni (Figure 4) can be explained by its strong correlation (As and Ni: *r* = 0.941, *P* < 0.01; As and Cr: *r* = 0.859, *P* < 0.01; Cr and Ni: *r* = 0.757, *P* < 0.01). This suggests that these metals may come from natural input or anthropogenic input. In this study, the elevated content of As, Cr, and Ni at certain station (Figure 4) is more likely to originate from anthropogenic sources. The highest concentration of Pb was found in LY 25 (6.99 *μ*g/L) while LY 3, LY 12, LY 16, and LY 21 were under the detection limits of ICP-MS (<0.005 *μ*g/L) with the mean value 1.07 ± 1.64 *μ*g/L. Langat River had become the main shipping route in transporting the products or materials from the factories located adjacent to the river. Leakages or spillages of leaded petrol from the boat or ship become one of the possible anthropogenic sources of Pb [9, 23]. However, all the sampling locations were below the MOH standard (10 *μ*g/L), and thus they could be classified as Pb-clean areas. The highest concentration of Co was in LY 1 (6.22 *μ*g/L) and the lowest in LY 24 (0.06 *μ*g/L). Co was possibly derived from lithogenic or anthropogenic sources, especially agricultural activities [38]. The mean concentration of Cd measured was 0.11 ± 0.12 *μ*g/L. The highest concentration was found in LY 3 (0.53 *μ*g/L), while LY 16, LY 21, and LY 22 were under the detection limits of ICP-MS (<0.01 *μ*g/L). All of the sampling locations were below the MOH standard (3 *μ*g/L), and thus they could be classified as Cd-clean areas. There was no significant correlation existing between Cd with other metals except for Pb (*r* = 0.325, *P* < 0.01). This relationship suggests that Cd is not specifically associated with other metals and was rather insignificant in the water metal distribution. Nevertheless, low concentration of metals in water might not necessarily reflect that the area was pollution-free. The metals in water tend to bind to sediment, and it might be accumulated from time to time and pose health hazard to aquatic biota [41].

### 3.3. Heavy Metals in Sediment

**Figure 5:**
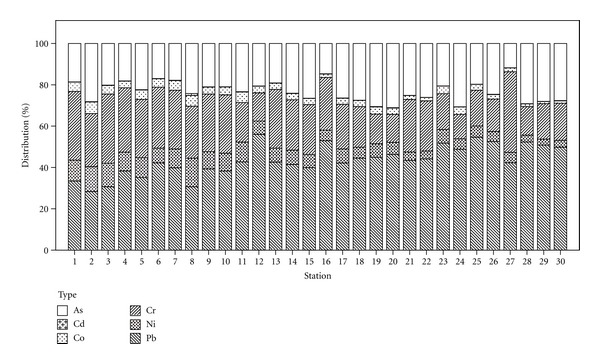
Distribution of metals in sediment samples from 30 sampling stations at Langat River.

The metal distribution in sediment follows the order: Pb > As > Cr > Ni > Co > Cd (Figure 5). One-way ANOVA analysis shows that metal concentrations were significantly different between stations (*P* < 0.05). This could be due to the variation of mineralogical composition in sediments from different stations or receive of different amounts of heavy metals that have been released from various sources [42]. Meanwhile, influence of river flow on sediment transport also plays a role in metal concentration. As the water current increases, the sediment particle is lifted into the water column and allows it to transport downstream toward the estuary. Therefore, a large part of the sediment may accumulate in the estuary and account for a higher level of metal concentration compared to upstream.

Table 6 represents the descriptive statistic for metal's concentrations in sediment. The results have been compared with the Canadian Interim Sediment Quality Guidelines (ISQG) and Probable Effect Levels (PELs) proposed by the Canadian Sediment Quality Guidelines for the Protection of Aquatic Life (Table 4) [25]. CCEM guidelines verified that only average value of As was higher than ISQG standard, which indicates that this metal is more likely to have adverse effects on organisms that live in sediment. The highest concentration was found in LY 29 (30.04 mg/kg) while lowest in LY 8 (4.47 ± 0.13 mg/kg). The average As concentration (16.19 ± 7.14 mg/kg) is measured to be roughly 10 times higher than the continental crust value of 1.70 mg/kg. About 97% of As value in sediment samples of the study area exceeded the ISQG standard (5.90 mg/kg) while 40% of these samples exceeded the PEL standard (17.00 mg/kg) [25].

**Figure 6:**
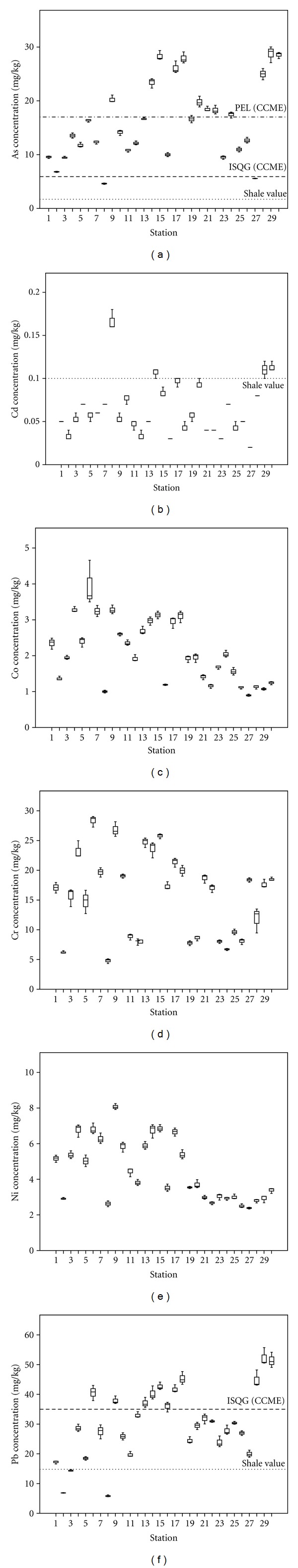
Distribution of each heavy metal concentration (mg/kg) along stations in Langat River sediments by comparing with average shale value and ISQG standard.

From Figure 6, it was observed that the highest concentration of Pb was found in LY 29 (55.71 mg/kg), where the steel-making factories were located, while the lowest was found in LY 8 (5.57 mg/kg). The average concentration of Pb (30.44 ± 11.77 mg/kg) was 2.1 times higher than continental crust value of 14.80 mg/kg. The present study revealed that around 37% of sediment samples was found to exceed the Pb-ISQG standard (35.00 mg/kg), and all samples were below the PEL standard (91.30 mg/kg) [25]. Table 6 shows that there was a positive correlation between Pb and a few elements that are often used in industries (As, Co, Cr, and Ni) which suggests that industrial activities were the source of Pb. Pb is toxic to aquatic organisms even at trace levels [43]. It also causes mental retardation among children, hypertension, intestinal cramps, peripheral nerve paralysis, anemia, and fatigue [2, 9, 44]. Higher concentrations of As and Pb were observed at upstream area compared with other metals (Figure 6). This might be because the mobility of these two elements in sediment was weaker compared with other metals such as Cr and Cd [45, 46]. Influence of metal input at the site near direct effluent discharged from industrial outlets is clearly seen by elevated contents of As and Pb [22, 27]. The metal's concentrations which exceeded the guideline values are critical enough to cause a toxic condition for aquatic organism. The highest concentration of Cr was found in LY 6 (29.04 mg/kg) while lowest in LY 8 (4.31 mg/kg). The average Cr concentration (15.88 ± 6.85 mg/kg) in sediment is lower than the continental crust value (126.00 mg/kg). The Cr concentration was possibly attributed to natural geological sedimentation of the area. However, the elevated value observed in this study may also be the result of anthropogenic activities [41]. The highest concentration for Ni was found in LY 9 (8.25 mg/kg) and the lowest in LY 8 (2.33 mg/kg). The average Ni concentration (4.47 ± 1.69 mg/kg) in sediment is lower than the continental crust value (56.00 mg/kg). It was observed that Cr was positively correlated with Ni (*r* = 0.744, *P* < 0.01), and this result corresponded with high Ni and Cr concentration in sediment at certain stations (Figure 6). The highest average concentration for Co (4.66 mg/kg) was found in LY 6 whereas the lowest (0.87 mg/kg) in LY 8. The average Co concentration (2.10 ± 0.86 mg/kg) in sediment is lower than the continental crust value (24.00 mg/kg). For Cd, the highest concentration was found in LY 8 (0.18 mg/kg) and the lowest in LY 27 (0.02 mg/kg). The average Cd concentration (0.06 ± 0.03 mg/kg) is lower than the continental crust value (0.15 mg/kg) while 13% of stations have exceeded the crust value. Compared with other metals, Cd and Co observed to be in low concentration and were consistent with the findings of Sarmani [22]. Meanwhile, the average concentration of As and Pb was found to be substantially higher than the metal concentrations from earth's crust value (Table 6). These values could be used to evaluate the load of anthropogenic metals from its surrounding. The results indicated that there is an anthropogenic metals-load in the river. The high concentration might also be attributed to the metal retention capacity of sediments as well as high industrialization activities within that area. The elevated concentrations of Pb, As, and Cr in the surface sediments of Langat River were toxic and may pose a hazard to the aquatic biota.

### 3.4. Correlation Coefficient between Metals and Physico-Chemical Properties

The correlation among the physico-chemical properties (pH and salinity) of water and sediment with different heavy metal concentrations was presented in Table 7. The correlation analysis was conducted to determine the relationship between metal concentration in water and sediment and the influence of physico-chemical parameters of water and sediment on metal's concentration. The pH and salinity are major concern in this study since they are the vital factors in metal solubility and control metals speciation and thus their distribution within dissolved fractions [43]. The present study shows that certain heavy metals were significantly correlated with these parameters in water and sediment.

The As, Cr, and Ni concentrations in water samples occurred a similar distribution pattern as depicted in Figure 4. These metal's concentrations in water were negatively correlated with pH sediment (*r* = 0.886, *P* < 0.01 for As, *r* = 0.762, *P* < 0.01 for Cr, *r* = 0.868, *P* < 0.01 for Ni) and conversely positively correlated with pH water (*r* = 0.494, *P* < 0.01 for As, *r* = 0.408, *P* < 0.01 for Cr, *r* = 0.322, *P* < 0.01 for Ni) as shown in Table 5. Under acidic condition, As and Cr exist as an anion is found to be rather immobile and tends to be sorbed by soil colloids [38, 47]. Such elements are more mobile in alkaline condition and tend to be released by soil colloid with increasing water pH. Similarly, pH sediment also positively correlated with Ni concentration in sediment itself (*r* = 0.385, *P* < 0.01). This indicated that as pH increased so did the binding of Ni to the water column or sediment solid phases. For Ni, increasing pH would reduce Ni mobility as insoluble hydroxides or carbonates are formed [47, 48]. Ni is easily sorbed by Fe and Mn oxides under high pH condition [47]. A similar result was observed by Doig and Liber [15] who reported that Ni complexation to organic matter was significantly influenced by pH. Hence, alkaline condition in pH sediment increased the concentration of As, Cr, and Ni in water. On the contrary, Co exhibited negative correlation with water pH (*r* = −0.377, *P* < 0.01), indicating an increase of Co solubility in water with decreasing in water pH. This phenomenon occurs as water pH decreases, Co complexes with organic and inorganic ligands tend to dissociate, and results in an increased free Co ions in water [38]. Furthermore, the competition between hydrogen ions and metal ions for binding sites on inorganic and organic ligands causes adsorption of Co by particulate matter decreases [47, 49]. Thus greater amounts of free Co ions in solution could release. Conversely, increasing in pH sediment increases the Co concentration in sediment with a correlation value (*r* = 0.340) due to the formation of insoluble carbonates or hydroxide [47, 48]. However, the *r* values between pH and metal are low despite the good significant level. According to Mackie [49] and Elzahabi and Yong [50], the solubility of heavy metals in soil water normally increases under acidic condition (pH <4). However, influence of pH on metal solubility is not obvious as the pH value in the present study ranged from 4.54 to 7.62.

Salinity also influences the solubility of As, Cr, and Ni in water. As the water salinity increases, the As, Cr, and Ni concentration in water was also increases (*r* = 0.963, *P* < 0.01 for As, *r* = 0.794, *P* < 0.01 for Cr, *r* = 0.898, *P* < 0.01 for Ni). Similarly correlation occurred between salinity of sediment and these metals (*r* = 0.903, *P* < 0.01 for As, *r* = 0.793, *P* < 0.01 for Cr, *r* = 0.882, *P* < 0.01 for Ni). This is because the increased salinity had increased the competition between cations and metals for binding sites on clay-organic particle surfaces, which interfere with the complexation and adsorption of metals [51]. Due to this competition relationship, more metals may be desorbed from the sediment and drive into the overlying water column.

## 4. Conclusion

The average concentration of studied metals (As, Cd, Co, Cr, Ni, and Pb) in the water was lower than Malaysian National Standard for Drinking Water Quality proposed by the Ministry of Health [24]. However, the river water was not suitable for drinking purposes due to the elevated concentration of studied metals at certain stations, especially As and Pb. The average values for As in sediment were exceeding ISQG standards as proposed by the Canadian Sediment Quality Guidelines [25]. This indicated that As in sediment has adverse biological effects with overexposure in Langat River. A comparison of the metal concentrations with average shale values reveals that most of the samples from the Langat River are polluted with Pb and As. Beside being originated from the geology of river bed and catchment area, the excess metal load in Langat River may also be attributed to the man-made activities such as discharge of industrial effluents and municipal wastes. The elevated concentration of certain metals in sediment posed a serious threat for the present and also the future because of their accumulative nature and toxicity effects toward organisms. This study provides valuable information on the environmental risk of metals in river water and sediments by comparison with quality guidelines. Spatial distributions of heavy metals in sediment and water have identified the vulnerable stations that are under threat. These results can provide a benchmark level to be used in the exploration of strategies to protect human health and the ecosystem.

## Figures and Tables

**Table 1:** The coordinate and notes of sampling stations.

Site	Latitude	Longitude	Notes
LY1	2^°^55^′^12.16^′′^N	101^°^25^′^49.98^′′^E	Village, near to mangrove area and palm oil plantation
LY2	2^°^54^′^18.20^′′^N	101^°^25^′^04.93^′′^E	Village, near to mangrove area and palm oil plantation
LY3	2^°^53^′^24.43^′′^N	101^°^24^′^48.25^′′^E	Village, near to mangrove area
LY4	2^°^52^′^31.16^′′^N	101^°^25^′^33.20^′′^E	Village, near to mangrove area, palm oil factory
LY5	2^°^53^′^25.51^′′^N	101^°^25^′^40.92^′′^E	Village, near to mangrove area, palm oil factory
LY6	2^°^54^′^26.50^′′^N	101^°^25^′^39.71^′′^E	Village, near to mangrove area, industrial activity
LY7	2^°^53^′^53.38^′′^N	101^°^26^′^33.14^′′^E	Village, near to mangrove area, industrial activity
LY8	2^°^53^′^28.28^′′^N	101^°^27^′^08.59^′′^E	Town, near to mangrove area, shipping activity, bridge connecting between Pulau Carey and Telok Panglima Garang
LY9	2^°^51^′^59.68^′′^N	101^°^26^′^23.10^′′^E	Near to mangrove area, the main river split into two estuary
LY10	2^°^52^′^28.37^′′^N	101^°^26^′^37.31^′′^E	Village, near to mangrove area, industrial activity
LY11	2^°^48^′^14.80^′′^N	101^°^24^′^26.80^′′^E	Village, near to mangrove area and Malacca Strait
LY12	2^°^50^′^23.60^′′^N	101^°^24^′^51.70^′′^E	Village, near to mangrove area
LY13	2^°^52^′^47.50^′′^N	101^°^27^′^06.00^′′^E	Village, near to mangrove area, shipping activity
LY14	2^°^53^′^23.10^′′^N	101^°^28^′^23.60^′′^E	Town, near to mangrove area, agricultural activity, village
LY15	2^°^52^′^32.20^′′^N	101^°^28^′^46.40^′′^E	Village, near to mangrove area, agricultural activity
LY16	2^°^51^′^19.50^′′^N	101^°^28^′^55.00^′′^E	Village, near to residential area and plantation area
LY17	2^°^51^′^10.70^′′^N	101^°^30^′^53.50^′′^E	Village, near to residential area and plantation area
LY18	2^°^49^′^51.30^′′^N	101^°^31^′^37.50^′′^E	Town, near to residential area and plantation area
LY19	2^°^48^′^47.60^′′^N	101^°^30^′^40.40^′′^E	Town, near to residential area and plantation area
LY20	2^°^48^′^47.30^′′^N	101^°^31^′^33.50^′′^E	Village, near to plantation area and residential area
LY21	2^°^48^′^41.20^′′^N	101^°^32^′^15.20^′′^E	Village, near to plantation area, new developed residential area
LY22	2^°^49^′^08.00^′′^N	101^°^33^′^02.10^′′^E	Near to plantation area and new developed residential area
LY23	2^°^49^′^04.80^′′^N	101^°^33^′^50.30^′′^E	Village, near to plantation area, industrial activity
LY24	2^°^49^′^00.30^′′^N	101^°^34^′^34.20^′′^E	Village, near to plantation area, industrial activity
LY25	2^°^48^′^41.40^′′^N	101^°^35^′^03.80^′′^E	Near to plantation area, massive industrial activity
LY26	2^°^47^′^56.40^′′^N	101^°^35^′^22.30^′′^E	Near to plantation area, massive industrial activity
LY27	2^°^48^′^04.80^′′^N	101^°^36^′^04.10^′′^E	Near to plantation area, massive industrial activity
LY28	2^°^47^′^43.50^′′^N	101^°^36^′^19.60^′′^E	Near to plantation area, massive industrial activity, outlet of industrial waste
LY29	2^°^47^′^27.38^′′^N	101^°^36^′^33.42^′′^E	Village, near to plantation area, industrial activity
LY30	2^°^47^′^25.20^′′^N	101^°^36^′^51.40^′′^E	Village, near to plantation area, industrial activity and part of agriculture activity

**Table 2:** Percentage recovery of metals for water analysis by ICP-MS.

Metals	Detection limit ranges (ppt)	Recovery (%)	Calibration curve (r)
As	1–10	102.29	0.9975
Cd	1–10	102.05	0.9999
Co	1–10	100.53	0.9999
Cr	1–10	101.53	0.9943
Ni	1–10	101.72	0.9996
Pb	<0.1–1	102.12	0.9999

**Table 3:** Average concentration (mg/kg) of metals in sediment analysis obtained from three replicates of the standard reference materials NIST, SRM 1646 (estuarine sediment).

Metals	Certified and estimated value	Measured value	Recovery (%)
As	6.23 ± 0.21^a^	5.73 ± 0.72	91.92
Cd	0.148 ± 0.007^a^	0.17 ± 0.05	111.59
Co	5.00^b^	4.96 ± 0.12	99.16
Cr	40.9 ± 1.9^a^	36.49 ± 1.89	89.23
Ni	23.00^b^	23.42 ± 0.79	101.84
Pb	11.7 ± 1.2^a^	13.01 ± 1.86	111.17

^a^Certified value.

^b^Estimated value.

**Table 4:** Descriptive statistic for selected matrices of water and sediment in Langat River.

	Unit	Minimum	Maximum	Range	Mean	SD	CV(%)
pH (w)	—	4.79	7.48	2.69	6.67	0.530	7.95
Salinity (w)	ppt	0.05	23.70	23.65	9.07	10.18	112.17
pH (s)	—	4.54	7.62	3.08	6.16	0.975	15.83
Salinity (s)	ppt	0.04	13.05	13.01	3.65	4.49	123.01

w: water, s: sediment, SD: standard deviation, CV: coefficient of variance.

**Table 5:** Descriptive statistic of heavy metal concentrations (*μ*g/L) in water samples at Langat River (*n* = 90).

	Minimum	Maximum	Range	Mean	SD	CV (%)	MOH (2004)
As	0.08	24.71	24.63	8.54	9.15	107.03	10.00
Cd	<0.01	0.53	0.53	0.11	0.12	103.79	3.00
Co	0.06	6.22	6.17	0.64	1.14	180.15	—
Cr	0.32	4.67	4.35	1.13	0.91	80.03	50.00
Ni	0.80	24.72	23.92	7.29	6.75	92.56	20.00
Pb	<0.005	6.99	6.99	1.07	1.64	153.25	10.00

SD: standard deviation, CV: coefficient of variance.

**Table 6:** Descriptive statistics of heavy metal concentrations (mg/kg) in sediment samples at Langat River (*n* = 90).

	Minimum	Maximum	Mean	SD	CV (%)	Average shale value	CCME	CCME
							(ISQG)	(PEL)
As	4.47	30.04	16.19	7.14	44.11	1.70	5.90	17.00
Cd	0.02	0.18	0.06	0.03	48.12	0.10	0.60	3.50
Co	0.87	4.66	2.10	0.86	41.51	24.00	—	—
Cr	4.31	29.04	15.88	6.85	43.15	126.00	37.30	90.00
Ni	2.33	8.25	4.47	1.69	37.94	56.00	—	—
Pb	5.57	55.71	30.44	11.77	38.66	14.80	35.00	91.30

SD: standard deviation, CV: coefficient of variance.

Average shale values (Wedepohl, 1995).

Interim Sediment Quality Guideline (ISQG) and Probable Effect Level (PEL) by CCME: Canadian Council of Ministers of the Environment; Canadian Sediment Quality Guidelines for the Protection of Aquatic Life (2002).

**Table 7:** Pearson correlation analysis between pH and conductivity with trace metals in water and sediment at Langat River, Selangor.

	pH (w)	Sal (w)	As (w)	Cd (w)	Co (w)	Cr (w)	Ni (w)	Pb (w)	pH (s)	Sal (s)	As (s)	Cd (s)	Co (s)	Cr (s)	Ni (s)	Pb (s)
pH (w)	1															
Sal (w)	0.669^∗∗^	1														
As (w)	0.494^∗∗^	0.963^∗∗^	1													
Cd (w)	−0.026	−0.062	0.002	1												
Co (w)	−0.377^∗∗^	0.236^∗^	0.384^∗∗^	−0.003	1											
Cr (w)	0.408^∗∗^	0.794^∗∗^	0.859^∗∗^	0.041	0.301^∗∗^	1										
Ni (w)	0.322^∗∗^	0.898^∗∗^	0.941^∗∗^	−0.029	0.603^∗∗^	0.757^∗∗^	1									
Pb (w)	−0.197	−0.323^∗∗^	−0.290^∗∗^	0.327^∗∗^	0.058	−0.210^∗^	−0.260^∗^	1								
pH (s)	0.501^∗∗^	0.894^∗∗^	0.886^∗∗^	−0.044	0.355^∗∗^	0.762^∗∗^	0.868^∗∗^	−0.393^∗∗^	1							
Sal (s)	0.529^∗∗^	0.907^∗∗^	0.903^∗∗^	0.045	0.415^∗∗^	0.793^∗∗^	0.882^∗∗^	−0.282^∗∗^	0.884^∗∗^	1						
As (s)	−0.281^∗∗^	−0.494^∗∗^	−0.527^∗∗^	0.169	−0.168	−0.456^∗∗^	−0.435^∗∗^	0.062	−0.354^∗∗^	−0.493^∗∗^	1					
Cd (s)	0.068	0.056	0.033	0.113	−0.018	−0.084	0.043	−0.001	0.104	−0.111	0.357^∗∗^	1				
Co (s)	0.207^∗^	0.409^∗∗^	0.370^∗∗^	0.087	0.253^∗^	0.227^∗^	0.475^∗∗^	0.114	0.340^∗∗^	0.313^∗∗^	0.242^∗^	0.028	1			
Cr (s)	−0.009	0.084	0.052	0.075	0.148	−0.065	0.180	−0.126	−0.011	−0.017	0.437^∗∗^	0.026	0.640^∗∗^	1		
Ni (s)	0.205	0.433^∗∗^	0.393^∗∗^	0.154	0.267^∗^	0.256^∗^	0.514^∗∗^	0.040	0.385^∗∗^	0.334^∗∗^	0.270^∗^	0.096	0.908^∗∗^	0.743^∗∗^	1	
Pb (s)	−0.266^∗^	−0.509^∗∗^	−0.571^∗∗^	0.068	−0.193	−0.565^∗∗^	−0.461^∗∗^	0.071	−0.422^∗∗^	−0.533^∗∗^	0.860^∗∗^	0.178	0.221^∗^	0.543^∗∗^	0.228^∗^	1

^∗∗^Correlation is significant at the 0.01 level (2-tailed).

^∗^Correlation is significant at the 0.05 level (2-tailed).
